# Editorial: Primary Antiphospholipid Syndrome

**DOI:** 10.3389/fimmu.2020.01993

**Published:** 2020-08-28

**Authors:** Antonio Serrano, Ricard Cervera, Jean Christophe Gris

**Affiliations:** ^1^Department of Immunology, Hospital Universitario 12 De Octubre, Madrid, Spain; ^2^Department of Autoimmune Diseases, Hospital Clínic, Barcelona, Spain; ^3^Department of Haematology, Centre Hospitalier Universitaire de Nîmes et Université de Montpellier, Nimes, France; ^4^I. M. Sechenov First Moscow State Medical University, Moscow, Russia

**Keywords:** PAPS, APS, thrombosis, B2GP1, PS/PT, cardiolipin

The antiphospholipid syndrome (APS) was described in 1983 as a systemic autoimmune disease characterized by the presence of thrombotic events or gestational morbidity in people carrying antiphospholipid antibodies (aPL). Although the disease was mainly detected in patients who already suffered from other autoimmune diseases, such as systemic lupus erythematosus (SLE), it was soon perceived that people with APS who did not suffer from other autoimmune diseases constituted a clearly differentiated clinical entity -the so called primary APS (PAPS) (1–3).

In recent years the interest of the scientific community on the APS has markedly grown. This e-book brings together basic, translational, and clinical research studies as well as reviews on the current situation of this disease.

The articles can be grouped into three blocks: New APS-associated biomarkers, pathogenesis and clinical associations, and reviews about specific aspects of the APS.

## New Biomarkers

In addition to patients with well-diagnosed APS, there are also asymptomatic people carrying aPL who never had an APS event (4). Conversely, there are patients with clear clinical APS criteria who are negative for the aPL included in the classification criteria, but may be positive for other aPL (5). It is very important to identify people at real risk to suffer APS events; therefore, the discovery of new biomarkers for diagnosis and monitoring the disease is an important aspect in this e-book.

López-Pedrera et al. address genomic/epigenetic changes related to the clinical profile of patients with APS and its modulation due to the effect of specific therapies.

Non-consensus anti-phosphatidylserine/prothrombin (aPS/PT), anti-domain 1 and anti-β2-glycoprotein I (aβ2GPI) antibodies of IgA isotypes have been discussed in several articles. McDonnell, Artim-Esen et al. determine the IgG subclass distribution for anti-domain1 and aβ2GPI antibodies. Litvinova et al. describe the potential value of aPS/PT antibodies as a strong marker of APS and propose that anti-PS/PT antibodies could be a surrogate APS biological marker of the lupus anticoagulant (LA) in patients whom LA detection cannot be achieved.

Sciascia et al. describe up to 45% of overall discrepant results for LA—and even higher in patients on vitamin K antagonists—and propose that the introduction of aPS/PT testing might represent a further diagnostic tool, especially when LA is not available or their results are uncertain. Serrano, Martinez-Flores et al. describe that antibodies of patients with thrombotic APS and IgA anti-β2GPI isolated positivity do not bind to domain 1 of β2GPI but bind other sites on domains 3 and 4 of β2GPI previously described as thrombosis-related epitopes.

Other non-aPL serum markers have been also studied. Manganelli et al. describe that serum levels of HMGB1 and sRAGE in APS patients are significantly increased when comparing to healthy subjects, highlighting that patients with APS and recurrent abortion showed significantly higher levels of sRAGE.

Serrano, Morán et al. studied patients with transplant-associated APS and show that pretransplant presence of circulating immune complexes predicts which aPL positive patients are at risk of thrombosis or early mortality after heart transplantation.

## Pathogenesis and Clinical Associations

In two articles a differential phenotype for the cells of the immune system that circulate in the blood is described in patients with APS. Lonati et al. describe that the percentage of blood cells with C4d in the membrane was significantly higher in patients with APS than in aPL negative controls, suggesting an important role of complement activation in APS. Álvarez-Rodríguez et al. found a less inflammatory profile in patients with PAPS that in SLE with higher levels of FoxP3 mRNA expression and reduced presence of circulating Th17 cells.

Palli et al. describe variations in type I interferon (INF) signature in APS patients. Type I IFN score is increased in PAPS and correlated positively with anti-β2GPI antibodies and negatively with hydroxychloroquine use.

Pérez et al. describe that the presence in the blood of APS patient of immune complexes of IgG/IgM bound to β2GPI is strongly associated with thrombocytopenia, leukopenia and complement consumption. In addition, these patients present non-criteria APS clinical manifestation, such as livedo reticularis and dry eyes.

McDonnell, Willis et al. propose a new therapeutic alternative using a PEGylated domain I of β2GPI pegylated. In laboratory assays, pegylated is capable of neutralizing IgG aβ2GPI of APS patients blocking its coagulopathic and thrombogenic properties.

## Reviews

Two revisions are also included in this topic. Kolitz et al. review APS cardiac damage, including treatment recommendations for each cardiac complication and Chaturvedi et al. review the role of complement in the pathophysiology of the APS and the use of its modulation in the treatment of catastrophic APS and thrombotic microangiopathy.

We hope that this collection of articles will help readers to better understand the characteristics of the APS. The progressive improvement of our knowledge about the origin, pathogenesis and mechanisms involved in the damage generated by the disease, together with the incorporation of new markers that help us to identify risks and assess the disease's staging, will help us to improve our therapeutic management and, ultimately, to help our patients in a more effective way.

## Author Contributions

AS, RC, and JG had a substantial contribution to the conception of the work, drafted the work, revised it critically and approved it for publication. All authors contributed to the article and approved the submitted version.

## Conflict of Interest

The authors declare that the research was conducted in the absence of any commercial or financial relationships that could be construed as a potential conflict of interest. The handling editor declared a past co-authorship with one of the authors RC.
